# Low-Concentration Hypochlorous Acid Drinking Water Alleviates Broiler Gut Microbial Load While Preserving Overall Growth Performance

**DOI:** 10.3390/toxics13010048

**Published:** 2025-01-10

**Authors:** Zonggang Li, Chang Liu, Dongyan Shao, Chune Tan, Yingqi Cao, Senzhong Deng, Teng Teeh Lim, Fei Xu

**Affiliations:** 1 Beijing Key Laboratory of Detection Technology for Animal-Derived Food Safety, College of Veterinary Medicine, China Agricultural University, Beijing 100193, China; s20203050772@cau.edu.cn (D.S.); s20223050922@cau.edu.cn (C.T.); caoyq@cau.edu.cn (Y.C.); 2 Key Laboratory of Agricultural Engineering in Structure and Environment, the Ministry of Agriculture and Rural Affairs of the People’s Republic of China, Beijing 100083, China; changliu2019@cau.edu.cn (C.L.); szdeng14@cau.edu.cn (S.D.); 3 Division of Plant Science and Technology, University of Missouri, Columbia, MO 65211, USA; limt@missouri.edu; 4 Key Laboratory of Feed Biotechnology, the Ministry of Agriculture and Rural Affairs of the People’s Republic of China, Beijing 100081, China

**Keywords:** drinking water quality, microbial quantity, drinking water additives, broiler gut, growth performance

## Abstract

Hypochlorous acid has been attempted as an additive to animal drinking water in practical animal farming processes for water microbial quality control. Despite its potential, there is still a knowledge gap concerning the effects of hypochlorous acid on both poultry growth performance and gut microbial load. To address this gap, an animal study was conducted using flow cytometry to quantify the age-related microbial load in broiler manure and gut contents. We observed that the effect on growth performance was sustained only during the starter phase, with no significant impact throughout the entire production cycle. The treatment could reduce the microbial load of both fresh broiler manure and cecal contents. Despite this convergence in the duodenum, significant differences in microbial loads between the control and treatment groups persisted in the manure and cecal contents throughout the later stages. Our findings demonstrate that consuming low-concentration hypochlorous acid water over the long term can lower the microbial load in the broiler gut throughout the entire growth cycle without impacting overall growth performance. Future research on drinking or feed additives should incorporate microbial absolute quantification methods to achieve a more precise assessment of microbiota.

## 1. Introduction

The measure of reducing antibiotic usage in food animal production has proven to significantly decrease antibiotic resistance in both animals and humans [1]. Consequently, sustainable alternative strategies and approaches to the healthy and efficient production of food animals in the post-antibiotic era are becoming increasingly imperative [2]. Meanwhile, current biosecurity management practices and technologies in food animal production are expected to encounter greater challenges and more stringent requirements [3,4]. Among these, maintaining the microbial quality of drinking water is a key component that plays a crucial role in the health, welfare, and production performance of food animals [5,6]. The formation and growth of biofilm on the inner surfaces of on-farm drinking lines, resulting from the accumulation of dissolved organic substances, minerals, and solid particles, can create a favorable environment for the attachment of opportunistic pathogens [7]. Moreover, the endpoints of the water distribution system in food animal farms, such as drinkers and nipples, may exhibit high levels of microbial contamination, even if the water source itself is of high quality [8,9]. Therefore, drinking water additives are employed during the raising period to prevent or inhibit biofilm formation and growth in the water distribution systems of food animal production [10,11]. Notably, additives used to improve drinking water quality will ultimately be ingested by food animals.

Hypochlorous acid (HOCl), the main component of slightly acidic electrolyzed water with a pH range of 5.0–6.5, derived from the electrolysis of diluted hydrochloric acid solution, has found growing use in food animal production as a cleaning product for microbial inactivation [12]. One potential application of HOCl water produced via electrolysis was to improve or maintain animal drinking water hygiene due to the overload of microorganisms [13,14]. Additionally, HOCl solution could be used as mouthwash liquid to control oral disease and maintain oral health [15,16]. This means that animals or humans could intentionally or unintentionally ingest some HOCl. Therefore, several animal studies were conducted to evaluate the systemic and gastrointestinal effects of ingesting HOCl. Seventeen mice were given unrestricted access to water containing HOCl for drinking purposes, and no anomalies were detected through visual examinations of the oral cavity, histopathological assessments, or evaluations of surface enamel roughness, indicating an absence of systemic effects [17]. Another animal study, which assessed serum aspartate aminotransferase, alanine aminotransferase, and creatinine levels and examined the main organs histopathologically under a light microscope in mice with access to slightly acidic electrolyzed water for 12 weeks, found no difference in functional and morphological health condition indices between the control and treatment groups [18]. Furthermore, HOCl was used as an additive to animal drinking water. Related research indicated that the addition of HOCl could not only improve drinking water quality by suppressing microbial reproduction but also play roles in food animal health maintenance [19,20,21]. The intestinal microbiota is universally recognized as a pivotal organ crucial for upholding host well-being, exerting a fundamental influence on diverse physiological processes [22]. However, the impact of HOCl as an additive in food animal drinking water on gut microbial load remains insufficiently understood.

The gastrointestinal regions in chickens host intricate microbial communities characterized by the coexistence of bacteria, fungi, viruses, archaea, and protozoa, with bacteria being the predominant taxonomic group [23]. Gut microbiota plays a key role in various aspects of both human and animal health in supporting food digestion, nutrient absorption, and immune function [24,25]. Within a larger context, current sequencing methodologies applied to fecal microbiota analysis quantify microbial taxa and metabolic pathways as relative abundances within the generated sample sequence library for each analytical procedure [26,27,28]. Although these comparative methodologies can enable the identification of microbiome variations associated with diseases, their capacity to unveil the intricate relationship between microbiota and host health is constrained [29]. An inherent constraint of sequencing technology is that the determined taxon abundances represent relative values [30]. Comparative analyses of relative microbiome data lack the capability to furnish details regarding the magnitude or directionality of alterations in taxa abundance or metabolic potential [31]. Therefore, careful consideration is necessary when interpreting these values biologically, as variations in cell density among samples are not taken into account [32]. Likewise, limited research related to the effect of HOCl on animal gut microbiota is based on the relative abundance of gut microbiota [20] or monoculture [33,34]. With the improvement in techniques and methodologies, several methods, including 16S qPCR, 16S qRT-PCR, ddPCR, reference spike-in, fluorescence spectroscopy, and flow cytometry, have been used to absolutely quantify the cell count of microbiota [35]. Although the existing strategies for the absolute quantification of microbiota exhibit considerable diversity and present notable challenges, the precise and highly sensitive quantification of total bacterial counts holds the potential for an unbiased interpretation of microbial dynamics and interactions.

To assess the effect of the long-term consumption of low-concentration hypochlorous acid water on broiler growth performance and the gut microbial load, we initially quantified the microbial load of broiler manure in both control and treatment groups at different weeks of age and subsequently measured the microbial load of different broiler gut content in both control and treatment groups at a matched age. Meanwhile, all growth performance parameters, including average daily feed intake, average daily gain, and the feed conversion ratio, were recorded for comparative analysis. A randomized controlled trial was used in this study to reduce bias and examine cause–effect relationships between the long-term consumption of low-concentration HOCl water and the gut microbial load. Flow cytometry, which was a quick, reproducible, and cost-efficient approach, was adopted in this study to quantify the microbial load. In addition, bacterial contamination in poultry farm water systems and the impact of hypochlorous acid addition were demonstrated based on published data [6,8,9,14,19,21,36,37].

## 2. Materials and Methods

### 2.1. Experimental Design

All broiler chicken procedures in this study were approved by the China Agricultural University Laboratory Animal Welfare and Animal Experimental Ethical Inspection Committee and complied with all relevant regulations (approval code: AW31103202-2-3, approval date: 13 January 2023). A total of one hundred and twenty newly hatched Arbor Acres broiler chicks were obtained from a commercial broiler hatchery and transported to the feeding facility within two hours. The one hundred and twenty broiler chicks were randomly and evenly divided into two groups. Each group included sixty broiler chicks in six replicates (ten broiler chicks housed in one pen were considered a replicate). The control group was provided with distilled water. The treatment group was provided with HOCl water during the full 42-day trial. The other environmental factors controlled in the laboratory feeding facility of the two groups followed the *Arbor Acres Broiler Management Handbook* (2018) [38]. All the broilers were fed a commercial diet consisting mostly of corn and soybean meal and provided free access to feed and water during the full 42-day trial.

The average daily feed intake (ADFI) and average daily gain (ADG) were monitored to assess the effect of HOCl water on the broilers’ production performance. All broiler chickens in each group were individually weighed on day 1 and then weekly throughout the entire trial. The weekly feed consumption for each pen was tracked. ADG, ADFI, and the feed conversion ratio (FCR; feed consumed/weight gain) were calculated for the periods of 1–14 days (starter phase), 15–28 days (grower phase), 29–42 days (finishing phase), and 1–42 days (entire cycle) [39].

### 2.2. Drinking Water Preparation and Dosage Determination

The waterline and nipple drinker were newly replaced before this experiment. Broilers in the control group and treatment group were provided free access to distilled water and HOCl water during the full 42-day trial, respectively. The free available chlorine (FAC) concentration of HOCl water used in this experiment was set to ~1 mg/L (pH = ~6.0), which was much lower than those (no observed adverse effect level) used in toxicology research [17,19,40] and can also suppress microbial reproduction in a poultry waterline [21]. The original HOCl solution (FAC = ~30 mg/L) was generated via the electrolysis of distilled water and hydrochloric acid electrolyte using an electrolyzed water generator (HD-240L, Shanghai Want Want Group, Shanghai, China). Then, the original HOCl solution was diluted using water with a pH of ~6.0 (distilled water with its pH adjusted via hydrochloric acid) until the FAC of the final diluted HOCl solution reached ~1 mg/L. The predominant free chlorine species was HOCl at a pH value of ~6.0 [41]. A digital chlorine assay kit (RC-3F; Kasahara Chemical Instruments Corporation, Saitama, Japan) was employed to analyze the FAC concentration, and a dual-scale pH meter (HM-30 R; DKKTOA Corporation, Tokyo, Japan) was utilized for the pH determination. The calibration of the pH meter was performed using commercial standard buffers supplied via the manufacturer, with pH values of 4.01 and 6.86.

### 2.3. Sampling

One chicken was randomly selected from each replicate in both the control group and treatment group on days 14, 28, and 42 to collect fresh manure. Although manure samples cannot reflect the real gut microbiome of a living animal comprehensively, it is still the most used and readily available. To further investigate the variation in the microbial load of the broiler gut, the selected chicken was then sacrificed for intestinal luminal sampling from the duodenum and cecum. The duodenum, the first intestinal segment of the broiler small intestine and the closest intestinal segment to the broiler gizzard, should be the initially affected intestinal segment of the lower digestive tract logically. The cecum exhibited richer microbial load and diversity compared to other parts, and it was also the second last segment of the broiler gut except the colon, which was short, and its inside content was close to the fresh manure. All the samples were divided into 0.2 g aliquots. One aliquot from two intestinal segments and fresh manure from each chicken were used immediately for cell count determinations.

### 2.4. Cell Count Detection

A 0.2-g aliquot of the sample was diluted 100,000-fold using a physiological solution containing 8.5 g/L NaCl. The diluted samples were filtered using a sterile syringe filter (pore size: 5 μm) to remove debris from the fecal solution. Then, each 1 mL of the microbial cell suspension obtained was stained with 1 μL of SYBR Green I (1:100 dilution in dimethyl sulfoxide; shaded 15 min incubation at 37 °C; 10,000× concentrate, Thermo Fisher Scientific, Waltham, MA, USA). A FACSCelesta flow cytometer (BD Biosciences, Franklin Lakes, NJ, USA) was used to perform the analysis of the microbial cell count present in the suspension, partly referring to a previously published method [42]. Fluorescence events were monitored using FL1 530/30 nm and FL3 > 670 nm optical detectors, and the forward- and sideways-scattered light was also recorded. Then, the same operation and analysis were conducted to quantify the background events with the liquid only without adding samples. The instrument and analysis were identical for all samples and background tests. The final fluorescence events were calculated by excluding the remaining background events to obtain an accurate microbial cell count.

### 2.5. Statistical Analysis

A *t*-test was employed to examine the mean differences between the control group and the treatment group at the same time point. The ANOVA was employed to explore differences among various time points in the same group. Normality and homogeneity of variance tests were conducted to ensure that the assumptions necessary for the applications of the *t*-test and ANOVA were met. IBM SPSS Statistics 24 (IBM corporation, New York, NY, USA) was used for the data analysis in this study.

## 3. Results

### 3.1. Bacterial Contamination in Poultry Farm Water System and the Impact of Hypochlorous Acid Addition

**Figure 1:**
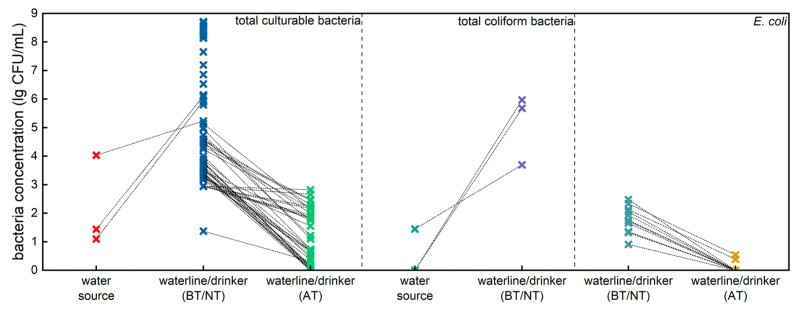
Distribution of bacterial contaminations in actual poultry production facilities and the effect of hypochlorous acid addition on bacterial concentrations’ reduction, as reported in published literature. Data sources: [6,8,9,14,19,21,36,37]. BT: before treatment; NT: no treatment; AT: after treatment by adding hypochlorous acid.

Figure 1 illustrates the distribution of bacterial concentrations in poultry farm water systems as reported in published literature [6,8,9,14,19,21,36,37]. The concentrations of total culturable bacteria and total coliform bacteria in the in-house waterlines or drinkers of poultry farms were much higher than those in the farm’s water source, indicating that bacterial contamination is occurring within the farm’s distribution system. Hypochlorous acid, a disinfectant effective against a broad range of pathogens, is produced through the electrolysis of diluted hydrochloric acid and has already been applied to poultry drinking water in actual production settings [14,19,21]. With the addition of hypochlorous acid, the concentrations of total culturable bacteria and E. coli in the in-house waterlines or drinkers of poultry farms were dramatically reduced. The results suggest that hypochlorous acid can be an effective treatment for reducing bacterial contamination in poultry farm water systems, thereby improving water quality and potentially enhancing poultry health and productivity.

### 3.2. Effect of Long-Term Consumption of Low-Concentration Hypochlorous Acid Water on Broiler Growth Performance

**Figure 2:**
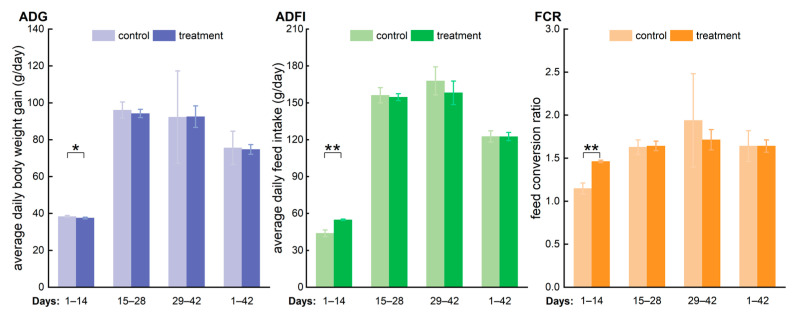
Growth performance of broiler chicken under the water-additive regimens of hypochlorous acid and the control. Average daily gain (ADG, n = 6), average daily feed intake (ADFI, n = 6), and feed conversion ratio (FCR, n = 6) (one-way ANOVA. * means *p* ≤ 0.05. ** means *p* ≤ 0.01.).

All broiler chickens remained healthy throughout the trial period. During the starter phase (days 1–14), significant differences in the average daily gain (ADG, *p* ≤ 0.05), average daily feed intake (ADFI, *p* ≤ 0.01), and feed conversion ratio (FCR, *p* ≤ 0.01) were observed between the control and treatment groups (Figure 2). Specifically, the treatment group exhibited a 2.0% lower ADG, a 20.0% higher ADFI, and a 21.5% higher FCR compared to the control group. However, during the grower (days 15–28) and finishing (days 29–42) phases, no significant differences were detected in ADG, ADFI, or FCR. Over the entire 42-day period, from birth to shelf, no significant differences in ADG, ADFI, or FCR were observed. These findings suggest that, while hypochlorous acid treatment influenced growth performance and feed efficiency during the early stages, its effects did not persist throughout the production cycle. This indicates that the initial impact of the treatment on broiler performance may diminish over time.

### 3.3. Effect of Long-Term Consumption of Low-Concentration Hypochlorous Acid Water on the Microbial Load of Fresh Broiler Manure

**Figure 3:**
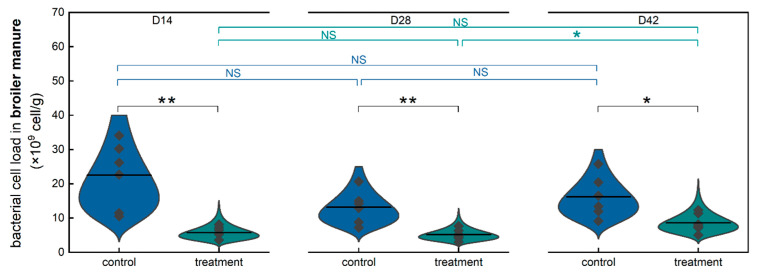
Comparative analysis of bacterial cell load of fresh broiler manure in control group and treatment group. (One-way ANOVA. * means *p* ≤ 0.05. ** means *p* ≤ 0.01. NS means no significance. D14, D28, and D42 mean day 14, day 28, and day 42, respectively. n = 6.).

Comparative analyses, including cross-sectional and longitudinal analysis, on the bacterial cell load of fresh broiler manure in the control and treatment groups were conducted to evaluate the long-term impact of consuming low-concentration HOCl water on the microbial load in fresh broiler manure at time points of day 14, 28, and 42 (Figure 3). In the context of cross-sectional comparison, the microbial load of fresh broiler manure in the treatment group was (5.73 ± 1.88) × 10^9^ cells/g at the time point of day 14, which was significantly lower than that in the control group (22.54 ± 9.72) × 10^9^ cells/g) (*p* ≤ 0.01). Similarly, the microbial load of fresh broiler manure in the treatment group was (5.20 ± 1.64) × 10^9^ cells/g at the time point of day 28, which was significantly lower than that in the control group (13.15 ± 4.81) × 10^9^ cells/g) (*p* ≤ 0.01). In addition, the microbial load of fresh broiler manure in the treatment group was (8.62 ± 2.73) × 10^9^ cells/g at the time point of day 42, which was significantly lower than that in the control group (16.22 ± 6.07) × 10^9^ cells/g) (*p* ≤ 0.05). In the context of a longitudinal comparison, the microbial load of fresh broiler manure presented no significant difference between the time points of day 14 and day 28 or day 14 and day 42 in the treatment group, while there was a significant difference (*p* ≤ 0.05) between the time points of day 28 and day 42 in the microbial load of fresh broiler manure in the treatment group. Meanwhile, no significant differences were observed between the time points of day 14 and day 28, day 28 and day 42, or day 14 and day 42 in the control group. The results indicated that the long-term consumption of low-concentration HOCl water can significantly reduce the microbial load of fresh broiler manure without consideration of microbial composition.

### 3.4. Effect of Long-Term Consumption of Low-Concentration Hypochlorous Acid Water on the Microbial Load of Broiler Duodenal Contents and Cecal Contents

**Figure 4:**
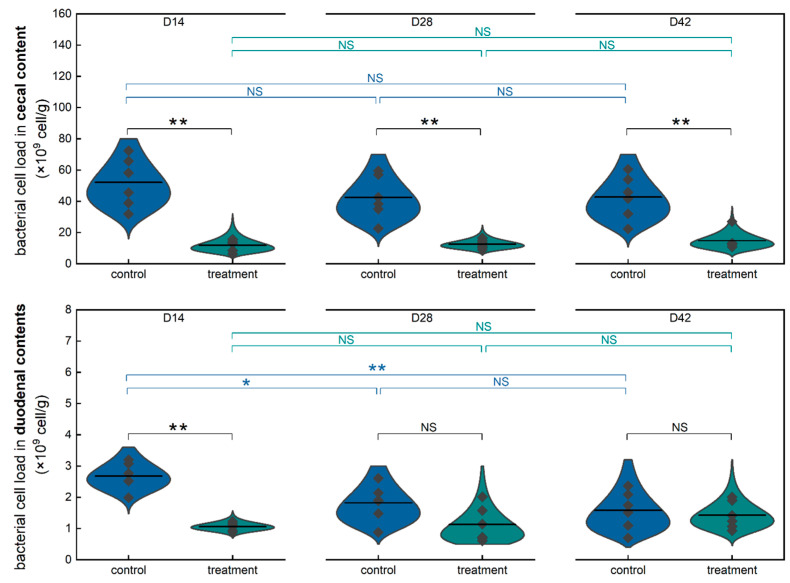
Comparative analysis of bacterial cell load of broiler cecal contents and duodenal contents in control group and treatment group. (One-way ANOVA. * means *p* ≤ 0.05. ** means *p* ≤ 0.01. NS means no significance. D14, D28, and D42 mean day 14, day 28, and day 42, respectively. n = 6.).

Furthermore, identical comparative analyses of the bacterial cell load of broiler cecal contents in control and treatment groups were conducted to investigate the long-term impact of consuming low-concentration HOCl water on the microbial load in broiler cecal contents at time points of day 14, 28, and 42 (Figure 4, cecal content). Similar differences were observed at the time points of day 14 and 28 in the context of cross-sectional comparison. The microbial load of broiler cecal contents in the treatment group ((11.91 ± 3.67) × 10^9^ cells/g) was significantly lower than that in the control group ((52.15 ± 15.84) × 10^9^ cells/g) (*p* ≤ 0.01) at the time point of day 14. Likewise, the microbial load of broiler cecal contents in the treatment group ((12.56 ± 2.87) × 10^9^ cells/g) was significantly lower than that in the control group ((42.56 ± 13.92) × 10^9^ cells/g) (*p* ≤ 0.01) at the time point of day 28. Notably, a significant difference in the microbial load of broiler cecal contents at the level of (*p* ≤ 0.01) was observed at the time point of day 48, which was unlike the difference (*p* ≤ 0.05) observed in the microbial load of the fresh broiler manure at the same time point. In the context of longitudinal comparison, the microbial load of broiler cecal contents presented no significant difference between the time points of day 14 and day 28, day 28 and day 42, or day 14 and day 42 in both control and treatment groups. The observations pointed to a significant reduction in the microbial cell load in broiler cecal contents caused by long-term, low-concentration HOCl water drinking.

To further examine the long-term impact of consuming low-concentration HOCl water on the microbial load of the initial segment of the intestine, identical comparative analyses of the bacterial cell load of broiler duodenal contents in the control and treatment groups were conducted (Figure 4, duodenal content). Obviously, the microbial load of the broiler duodenal contents was much lower (around one order of magnitude) than those of the fresh manure and cecal contents. In the context of cross-sectional comparison, the microbial load of broiler duodenal contents in the treatment group ((1.06 ± 0.13) × 10^9^ cells/g) was significantly lower than that in the control group ((2.68 ± 0.44) × 10^9^ cells/g) (*p* ≤ 0.01) at the time point of day 14, which was similar to those in fresh manure and cecal contents. However, in contrast with the observations in fresh manure and cecal contents, the microbial loads of broiler duodenal contents in the treatment group ((1.13 ± 0.57) × 10^9^ cells/g at Day 28, (1.43 ± 0.44) × 10^9^ cells/g at Day 42) have no significant difference with those in the control group ((1.82 ± 0.59) × 10^9^ cells/g at Day 28, (1.59 ± 0.62) × 10^9^ cells/g at Day 42), respectively. In the context of longitudinal comparison, the microbial load of broiler duodenal contents presented no significant difference between the time points of day 14 and day 28, day 28 and day 42, or day 14 and day 42 in the treatment group. Similarly, the microbial load of broiler duodenal contents presented no significant difference between the time points of day 28 and day 42 in the control group. Meanwhile, there was a significant difference (*p* ≤ 0.05) between the time points of day 14 and day 28 in the microbial load of broiler duodenal contents in the control group. Additionally, there was a significant difference (*p* ≤ 0.01) between the time points of day 14 and day 42 in the microbial load of broiler duodenal contents in the control group. It is evident from the results that the consumption of low-concentration HOCl could significantly reduce the microbial load of broiler duodenal contents at the beginning (day 14); afterward, no impact was observed on the microbial load of broiler duodenal contents (day 28 and day 42). Interestingly, the microbial load in the control group decreased as the broiler age increased and eventually reached the same level as the treatment group in the later period. However, significant differences in microbial loads between the control group and the treatment group persisted in the later period.

## 4. Discussion

Hypochlorous acid (HOCl), currently being explored as a potential water additive in poultry production to enhance drinking water quality, will ultimately be consumed by poultry. The digestive system and its symbiotic gut microbiota are likely to be the first to be affected. Meanwhile, flow cytometry has emerged as a proposed method for the absolute quantification of microbiota, addressing the limitations of culture-based plate counts and the relative data obtained from gene sequencing [27,32,42,43,44,45,46]. In our study, we presented the distribution of bacterial concentrations in poultry farm water systems based on published literature and then investigated the effects of the long-term consumption of low-concentration hypochlorous acid water on the average daily feed intake (ADFI), average daily gain (ADG), feed conversion ratio (FCR), as well as the microbial load in broiler fresh manure and intestinal content. We found that bacterial concentrations at the endpoints of the poultry drinking water line were significantly higher than those at the water source. Moreover, the addition of HOCl significantly reduced bacterial concentrations at these endpoints compared to the control group, as supported by current limited published research. Notably, hypochlorous acid treatment influenced growth performance and feed efficiency during the early stages, while its effects did not persist throughout the entire production cycle. Regarding microbial load variation, long-term consumption of low-concentration HOCl water could maintain the microbial load in broiler manure and intestines at a lower level (within a fivefold difference), independent of variations in microbial composition.

In a previous study, incorporating slightly acidic electrolyzed water with an available chlorine concentration of 0.3 mg/L into the daily management of the drinking water system for laying hens suggested a higher fecal normal rate [21]. In another animal experiment, basic electrolyzed water (pH = 9.90) was given to mice as drinking water for four weeks. The 16S rRNA gene sequencing analyses of mouse fecal samples revealed notable differences in the relative abundances of 20 taxa among mice administered with basic electrolyzed water [47]. Limited studies indicated that drinking low-concentration hypochlorous acid water may have an impact on the animal fecal microbiota. The hypochlorous acid solution provided as drinking water to rats was found not to completely eradicate *p. aeruginosa* from the rats but, rather, to be effective in preventing infection without adversely affecting serum biochemical variables using a culture-based method [40]. Additionally, several studies on chlorinated drinking water, along with relative microbiome data, have indicated alterations in animal gut microbiota [48,49,50]. However, there is a knowledge gap concerning the effect of the long-term drinking of low-concentration hypochlorous acid water on the animal fecal microbial load. Our study revealed that the long-term drinking of low-concentration hypochlorous acid water can keep the microbial load of fresh broiler manure at a lower level, given that the manure microbiota could only predict a fraction of the microbiota at most gut sites [51]. Our research findings further demonstrate that the continuous consumption of low-concentration hypochlorous acid water effectively lowers the microbial load at both the duodenum and cecum. Drinking low-concentration hypochlorous acid water could suppress the microbial load in the animal gut, which is partly similar to the administration of low-level antibiotics to some extent.

We acknowledge limitations yet also future challenges in our study. Only the changes in microbial load of the broiler gut and manure were investigated, without considering the variations in microbial composition. It is hard to conclude that microbial load reduction is detrimental to animals without a comprehensive assessment. For example, antibiotic growth promoters, described as any medicine that destroys or inhibits bacteria and is administered at a low, sub-therapeutic dose, are used to help growing animals digest their food more efficiently, get the maximum benefit from it, and develop into strong and healthy individuals [52]. Thus, more rigorous laboratory animal experiments are necessary to investigate the effects of long-term hypochlorous acid water drinking at a low concentration on broiler feed conversion, toxicology, disease resistance, gut microbial composition, etc. Additionally, via the oral intake of the hypochlorous acid water, it is expected that there will be an impact on the microbiota in the upper part of the broiler digestive tract. However, all samples in our study were collected from the lower part of the broiler digestive tract (duodenum, cecum, and fresh manure). Generally, the oral route is one of the primary pathways for environmental pathogens to enter the body. Theoretically, the consumption of low-concentration hypochlorous acid water could reduce the retention quantity of pathogens in the upper digestive tract, thereby reducing the disease infection risk via the oral route.

## 5. Conclusions

Our findings demonstrated that the long-term consumption of low-concentration hypochlorous acid water had no impact on overall growth performance throughout the production cycle. The microbial load of fresh broiler manure was significantly reduced by the long-term consumption of low-concentration hypochlorous acid water throughout the whole broiler growth cycle. Furthermore, the animal study on the long-term consumption of low-concentration hypochlorous acid water showed that the microbial load of broiler cecal contents underwent a significant reduction during the whole broiler growth cycle. Additionally, as the broiler age increased, the microbial load of the broiler duodenal contents in the control group decreased and eventually aligned with that of the treatment group in the later stages. Despite this convergence, notable disparities in both the microbial loads of the fresh broiler manure and the broiler cecal contents between the control and treatment groups persisted throughout the later period. The findings of this study warrant that long-term hypochlorous acid water drinking at a low concentration could result in a decrease in the microbial load of the broiler gut during the whole broiler growth cycle without affecting the overall growth performance. Additionally, future experimental research on feed or drinking additives should employ microbial absolute quantification methods to better assess microbiota.

## Data Availability

The data that support the findings of this study are available from the co-corresponding author, Zonggang Li, upon reasonable request.
